# Mechanistic understanding of perianth traits hindering pollination in *Aristolochia contorta* Bunge

**DOI:** 10.3389/fpls.2023.1226331

**Published:** 2023-09-21

**Authors:** Si-Hyun Park, Jae Geun Kim

**Affiliations:** ^1^ Department of Biology Education, Seoul National University, Seoul, Republic of Korea; ^2^ Center for Education Research, Seoul National University, Seoul, Republic of Korea

**Keywords:** *Aristolochia contorta* Bunge, northern pipevine, sexual reproduction, trap flower, perianth function, pollinator, Ceratopogonidae spp.

## Abstract

Insects are vital pollinators for angiosperms, playing a crucial role in their reproductive success and fruit production. *Aristolochia contorta* is a perennial herbaceous vine that occurs in fragmented populations across East Asia. One notable feature of this plant is its trap flower, which employs a unique mechanism to attract, trap, retain, and release insects, ensuring effective pollination. The presence of this trap flower significantly influences the pollination system of *A. contorta.* Field surveys and pollination experiments were conducted to understand the processes and effectiveness of its pollination mechanism. It was allogamous and was pollinated by the species from Ceratopogonidae. During the insect attraction stage, 11.57% of the flowers contained insects, primarily Ceratopogonidae spp. Most Ceratopogonidae spp. concentrated in few flowers, indicating that although overall attraction might be modest, specific flowers acted as significant focal points for gathering. Trichomes effectively trapped Ceratopogonidae spp. inside flower tubes. In the retention stage, 26.16% of Ceratopogonidae spp. were loaded with pollen grains, but only 7.91% of those exited the flowers in the release stage. The sticky texture of the perianth’s internal cavity posed challenges during this release, leading to adhesion and clogging of the narrow perianth tube. Consequently, a significant portion of Ceratopogonidae spp. became trapped on the perianth wall and perished. This highlights that despite the significant energy and resources invested in flower development, the perianth contributes to the low pollination effectiveness. This study revealed additive factors with negative effects on pollination, including the densely clustered distribution of its pollinators within only a few flowers, insufficient pollen loading onto pollinators, hindered release of entrapped pollinators due to the perianth adhesive surface, and a high rate of defective pollen grains in *A. contorta*. These factors account for the observed phenomenon of low fruit set (7.7%) and contribute to the diminished rate of sexual reproduction in *A. contorta* populations. This might lead the species to heavily rely on asexual reproduction, which could potentially lead to gene erosion within populations. The implications of these findings extend to the ecological and conservation aspects, emphasizing the need to understand and conserve the unique pollination system of *A. contorta*.

## 1 Introduction

Angiosperms exhibit diverse pollination mechanisms, with insects serving as their primary pollinators. This insect-mediated pollination is believed to significantly contribute to the reproductive success of angiosperms and their fruit production (Burger, 1981). Pollinators promote cross-pollination by transporting pollen from one flower to another, leading to enhanced genetic diversity. Despite the presence of pollinators, in some cases, certain angiosperms may experience low fruit set, prompting inquiries into the underlying factors that influence fruit set in these plants (Ashman et al., 2004). Previous research has explored various factors such as environmental conditions, genetic factors, reproductive barriers, and resource availability, to understand the causes of poor fruit set (Knight et al., 2005; Sukhvibul et al., 2005; Gebremariam and Tesfay, 2019).

Flowers of the Aristolochiaceae family have evolved the ability to cross-pollinate. However, some members of the family, such as *Aristolochia serpentaria* L., can employ self-pollination as an alternative strategy (Pfeifer, 1966). The genus *Aristolochia* is the first group of angiosperms to develop a highly complex and specialized pollination mechanism (attracting, trapping, retaining, and releasing insects) that may have been designed to attract and trap particular pollinators (Oelschlägel et al., 2009; González et al., 2014). The shape and color of the flowers of numerous *Aristolochia* species have evolved to trap small pollinators from various dipteran families (Aliscioni et al., 2017; Martin et al., 2017), including Calliphoridae, Ceratopogonidae, Ophilidae, Mycetophilidae, and Phoridae (Berjano et al., 2009; Hipólito et al., 2012). Moreover, *Aristolochia* flowers exhibit a sapromyiophilous or micromyiophilous pollination mechanism mediated by Diptera (Vogel, 1990; Johnson and Jürgens, 2010). In addition to depending on dipteran pollinators, the reproductive success of *A. contorta* relies on various factors, including the availability and efficiency of pollinators, floral traits, pollen availability and quality, breeding system, resource availability, abiotic factors, as well as interactions with herbivores and pathogens (Herrera, 2000; Knight et al., 2005; Lucas-Barbosa et al., 2011; Rafferty and Ives, 2012). The flowers of plants in the genus *Aristolochia* display protogyny, where the gynoecium of the flower matures before its androecium, preventing self-pollination (Murugan et al., 2006; Oelschlägel et al., 2009). Depending on the maturation of the female and male parts, morphological changes in the flower cause a transition to insect release during pollination (Gorb and Gorb, 2002). Despite these remarkable strategies, some *Aristolochia* species still experience challenges in achieving optimal fruit set. The poor fruit set observed in certain *Aristolochia* species can be attributed to multiple factors. One significant factor is the intricate timing and coordination required for successful pollination, with protogyny and specific interactions with insect pollinators playing a crucial role (Razzak et al., 1992; Oelschlägel et al., 2016). Furthermore, the availability and effectiveness of pollinators, particularly small flies, can influence the pollination process and lead to reduced fruit set (Rulik et al., 2008; Aliscioni et al., 2017). Recent studies examining *Aristolochia* spp. in various ecological settings have contributed to our understanding of pollination mechanisms and adaptations within the Aristolochiaceae family. For instance, *A. bianorii* was explored in island ecosystems (Alpuente et al., 2023), *A. chilensis* in arid environments (Stotz and Gianoli, 2013), and *A. debilis* in central Japan (Sugawara et al., 2016). Collectively, these studies enhance our knowledge of the fascinating pollination dynamics and adaptations within the Aristolochiaceae family.

The perennial herbaceous, twining-stem, ground-rooted vine species *A. contorta* Bunge (Aristolochiaceae) is neither parasitic nor epiphytic and is found in fragmented natural populations in East Asia. It exhibits both asexual and sexual reproduction (Lee, 2012). However, the genetic diversity of *A. contorta* in South Korea is very low compared to that observed in Russia, China, or Japan, suggesting that it relies on asexual reproduction (Nakonechnaya et al., 2012; Nam et al., 2020). The larvae of the butterfly *Sericinus montela*, designated as a vulnerable species on a regional scale in South Korea according to IUCN criteria, only feed on *A. contorta* (Lee, 2012). Studies on the effectivity of pollination mechanisms are required to ensure conservation and genetic diversity in *A. contorta.*

Studies on the ecological significance of *A. contorta* have focused on functional aspects, such as plant secondary metabolites and optimal habitat conditions; for instance the presence of aristolochic acids (Hashimoto et al., 1999; Cheung et al., 2006), local uses and nephrotoxic properties (Heinrich et al., 2009), reproductive traits and productivity (Nakonechnaya et al., 2013), the optimal habitat for conservation (Park et al., 2019), and climate change impacts on plant growth and secondary metabolites (Park et al., 2021; Park et al., 2022). These studies contribute to our understanding of the chemical composition, ecological interactions, reproductive biology, and conservation strategies of *A. contorta*. However, research on the factors contributing to the low sexual reproduction rates observed for this species in Korea is lacking. In particular, there have been no studies on the reproduction of *A. contorta* or the relationship between *A. contorta* and its pollinators.

This study aimed to examine the pollination mechanisms of *A. contorta*. To that end, we investigated the factors contributing to the low fruiting rate, with a specific focus on low pollination effectiveness, and to capture the intricate ecological dynamics of flower-pollinator interactions in their natural context. Therefore, during this study we investigated the effectiveness of the pollination mechanism of *A. contorta* through field surveys and pollination experiments. The surveys and experiments provided new data on the flower morphology, pollinators, and pollination mechanism of *A. contorta*. These findings enhance our understanding of the pollination mechanism of *A. contorta*, as well as the role of perianth and pollinators in this process. By assessing the impact of pollinator availability and efficiency, as well as the role of floral traits, our study focused on the intricate dynamics influencing the reproductive success of *A. contorta*. Furthermore, this study will contribute to the field by uncovering the mechanisms behind pollination effectiveness, its influence on reproductive success, and providing insights into the genetic diversity of *A. contorta*.

## 2 Materials and methods

### 2.1 Study site

The research was conducted in Anyang, Gyeonggi Province, South Korea (37°24′2.58 ′′ N, 126°58′18.3′′ E) from June 18 to August 27, 2022, which was the flowering period of *A. contorta*. During the research period, temperatures ranged from 22.2°C to 35.8°C. This site was located in a riparian area within the native distribution range of *A. contorta*.

### 2.2 Characteristics of flowers

**Figure 1:**
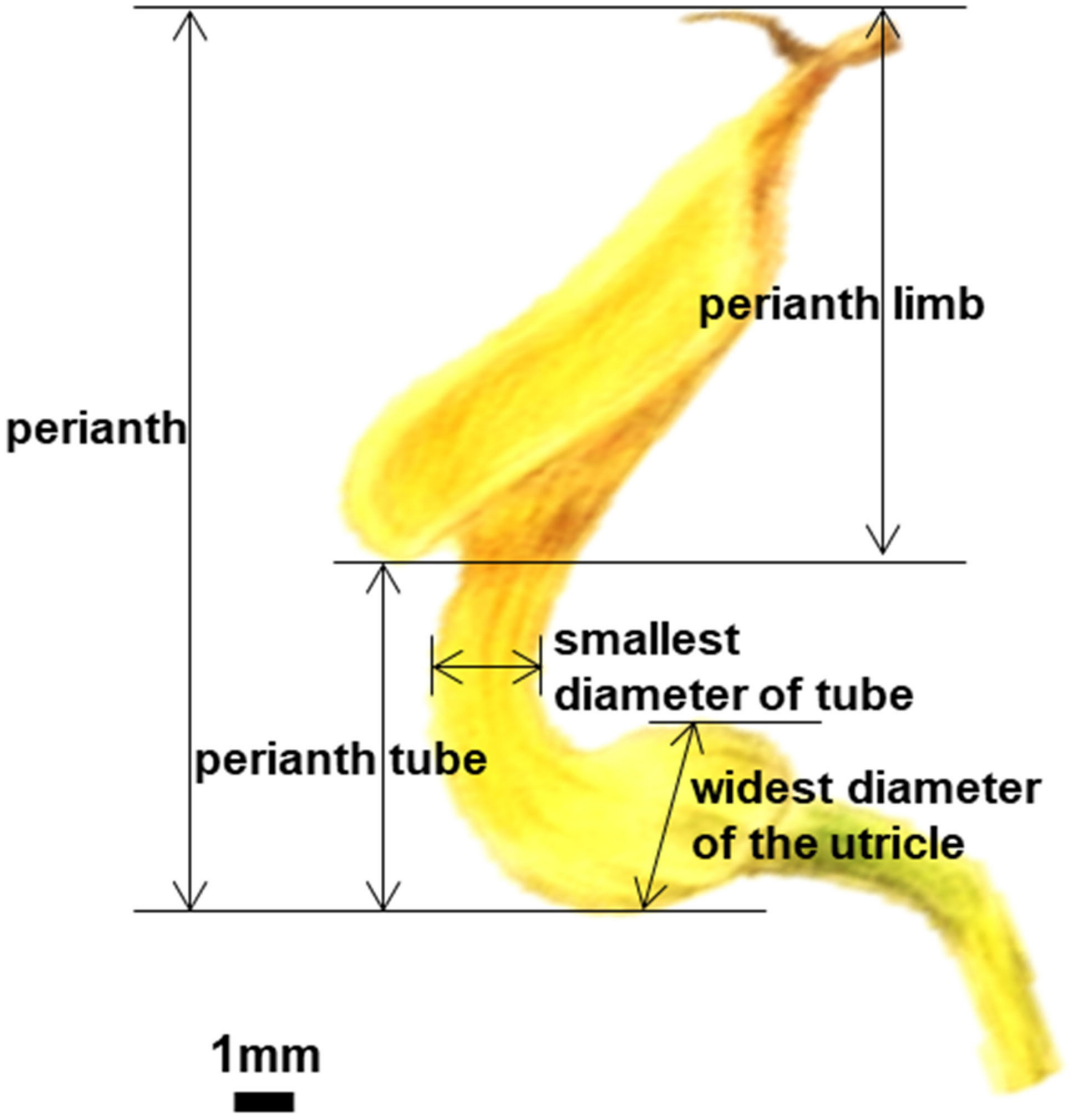
Structural measurement of perianth of *Aristolochia contorta*.

To obtain a broad perspective on the mechanical trapping of pollinators achieved by the morphological fit of the floral parts, we observed the blooming order of flowers from the same leaf axil (based on wilting order), flower color, the structure of floral elements, and days of developmental phases of the reproductive organs in *A. contorta*. Additionally, the perianth length, perianth tube length, and smallest diameter of the tube (i.e., constriction of the tube above the basal inflation and small openings at the topmost part of the perianth tube in *A. contorta*), as well as the widest diameter of the utricle from each flower of ten individual plants were measured using vernier calipers (**Figure 1**). Hydrogen peroxide (H_2_O_2_) solution (3%) was placed on the stigmas of the female phase and male phase respectively and the appearance of bubbles was observed for 3 mins (Dafni and Maués, 1998; Serrano and Olmedilla, 2012). The receptivity was measured based on the level of bubble formation observed. If a stigma produces a significant amount of bubbles, it is considered to have a high reactivity to the compound, while little to no bubble formation, indicates that it has low reactivity.

### 2.3 Insect visitation

To monitor flower visitors, close-up videos of 3–10 flowers (the flowers of a different individual every week) were recorded three times a day, on 1 day per week, for 10 weeks (48.67 m/session, a total of 73 h of recording). Data on flower visitors were collected at weekly intervals from June 18, 2022, when the flowers began to bloom, to August 28, 2022, when all flowers had wilted. Simultaneously, we sampled less than 10% of the flowers in the ten random quadrats (as *A. contorta* has been designated as a vulnerable species, less than 10% of the total flowers were collected). Each quadrat had a range of 3 to 8 individuals. We collected flowers three times a day to determine visitor species; during the post-sunrise period (1,590 flowers), in daytime (1,619 flowers), and directly post-sunset (1,349 flowers). Furthermore, we collected flowers during the female (686 flowers) and male phases (3,487 flowers), as well as when the flowers were wilted (629 flowers). We also collected samples from plants that had a height of more than 1 m (2,806 flowers), or a height of under 1 m (1,752 flowers), and flowers at different temperatures such as 22°C-27°C (1,191 flowers), 27°C-32°C (2,738 flowers), and 32°C-36°C (629 flowers). Subsequently, the same set of flowers was used in different subsets for distinct comparisons.

To investigate whether pollinators easily emerge when transitioning from the female flower phase to the male flower phase, similar to the behavior observed in pollinators of other *Aristolochia* plants, the collected flowers were stored upright in separate 100 mL plastic bottles, mimicking the original flower position, for 48 hours. This period allows sufficient time for the loss of turgidity and the contraction of trichomes in the perianth tubes, which facilitate the emergence of insects (Oelschlägel et al., 2009; González and Pabón-Mora, 2015). Therefore, 48 hours post flower collection, all visitors found inside or outside the flowers were fixed in 70% ethanol and the number of visitors in the flower bottle were recorded. Subsequently, the individuals were identified to the family or genus level, and grouped into morphospecies using taxonomic keys by Lee (2004). The flowers were then opened with forceps, and the presence of the remaining flower visitors was assessed under a stereoscopic microscope (Digital Microscope, GB-742, Global4U, Republic of Korea). The species and the number of insects (including dead insects) were recorded according to the phase of all flowers, time, and height. In addition, the body lengths (measured from the tip of the antennae to the end of the abdomen) and widths (measured at the thickest part of the body) of potential pollinators were measured. We considered potential pollinators as those that have pollen grains on their bodies and a high chance of coming into contact with the pollen of a flower, thereby facilitating its transfer to another flower and aiding in the reproductive process.

### 2.4 Pollination experiment

Three groups were used to test the probability of autogamy. In the first group, 211 randomly selected closed flower buds from 10 individuals were enclosed in fine-mesh bags (mesh size 0.5 × 0.65 mm) before the onset of anthesis to prevent insect visitation and left in the enclosed bag for a month (Tarachai et al., 2008; Blaauw and Isaacs, 2014). Similar to other studies on *Aristolochia* species (Sakai, 2002; Oelschlägel et al., 2009), we investigated the role of the perianth chamber in the pollination mechanism to determine whether it played a crucial role. To test the function of the perianth chamber, the perianth tubes and antheses of 194 randomly selected closed flower buds were removed from 10 individuals in the second group (all flowers with opened perianths were removed). In the third group (control group), 206 flower buds were randomly selected from 10 individuals and were marked. Following that, the identified flower buds were intentionally left exposed to pollinators without any protective covering or bagging. After one month, the percentage ratio between the number of fruits in the three groups was measured (Percentage of Fruit Set = (Number of Fruits/Number of Flowers) x 100).

### 2.5 Pollen distribution on pollinator bodies

The pollination effectiveness of a pollinator depends on its ability to transfer pollen grains between flowers, by removing and depositing them during the process. We focused on the initial stage of pollination, which involves the removal of pollen grains (Jones, 2012). After opening 4,558 flowers with precision forceps, the number of visitors carrying pollen grains and the number of pollen grains loaded onto the insects were counted. Using quantitative palynology, we employed a gridded slide to create a quantification system for counting pollen grains (Jones, 2012). The pollen grains were observed and counted under both a stereoscopic microscope (Digital Microscope, GB-742, Global4U, Republic of Korea) and an optical microscope (OS-N1000DM, Osunhitech, Republic of Korea). At 48 hours post opening, the majority of flowers would have transitioned to the male phase (Berjano et al., 2009). Given the numerous variables associated with pollen loading or loss, our study specifically focused on the pollen actively loaded by pollinators when we opened the flowers at the 48 hours post opening time point. The locations of pollen grains attached to floral organs and pollinators were observed using scanning electron microscopy (SEM). For SEM, 10 A*. contorta* flowers and 25 pollinators with pollen grains on their body were collected. The gynostemium was cut with a razor, dividing it into stigma and stamen. In addition, the ovary was cut into cross-sections with a razor to enable the observation of the embryos. Subsequently, they were fixed with osmium tetroxide (OsO_4_) and dehydrated using a series of alcohol solutions (30%, 50%, 70%, 90%, and 100%, Ul-Hamid, 2018). Next the samples were subjected to critical-point drying with liquid CO_2_ (Critical Point Dryer, EM CPD300, Germany). Double-sided copper tape was applied onto the stubs, and materials were mounted appropriately to securely hold the samples (Ul-Hamid, 2018). To avoid accidental captures, the materials were then sputter-coated with platinum (Sputter Coater, EM ACE200, Austria) and examined using a Field-Emission Scanning Electron Microscope (FESEM, SIGMA, Carl Zeiss, UK) at an accelerating voltage of 30 kV at the National Instrumentation Center for Environmental Management (NICEM) at Seoul National University.

### 2.6 Data analysis

To determine if there are significant differences in the fruit set percentages between groups in the pollination experiment, a non-parametric analysis using the Kruskal-Wallis test was conducted. The statistical software SPSS ver. 23.0 (SPSS, Inc., Chicago, IL, USA) was utilized for the analysis, and the significance level was set at p < 0.05. Variations in the distribution of visitors according to different conditions [the time of day (sunrise, daytime, sunset), dissimilar developmental stages (female phase, male phase), height (more than 1 m, less than 1 m), temperature (22°C–27°C, 27°C–32°C, 32°C–36°C)] were analyzed with multivariate ANOVA (MANOVA) using SPSS ver. 23.0 software (SPSS, Inc., Chicago, IL, USA). Significance level was set at p < 0.05.

## 3 Results

### 3.1 Morphology of flowers

**Figure 2:**
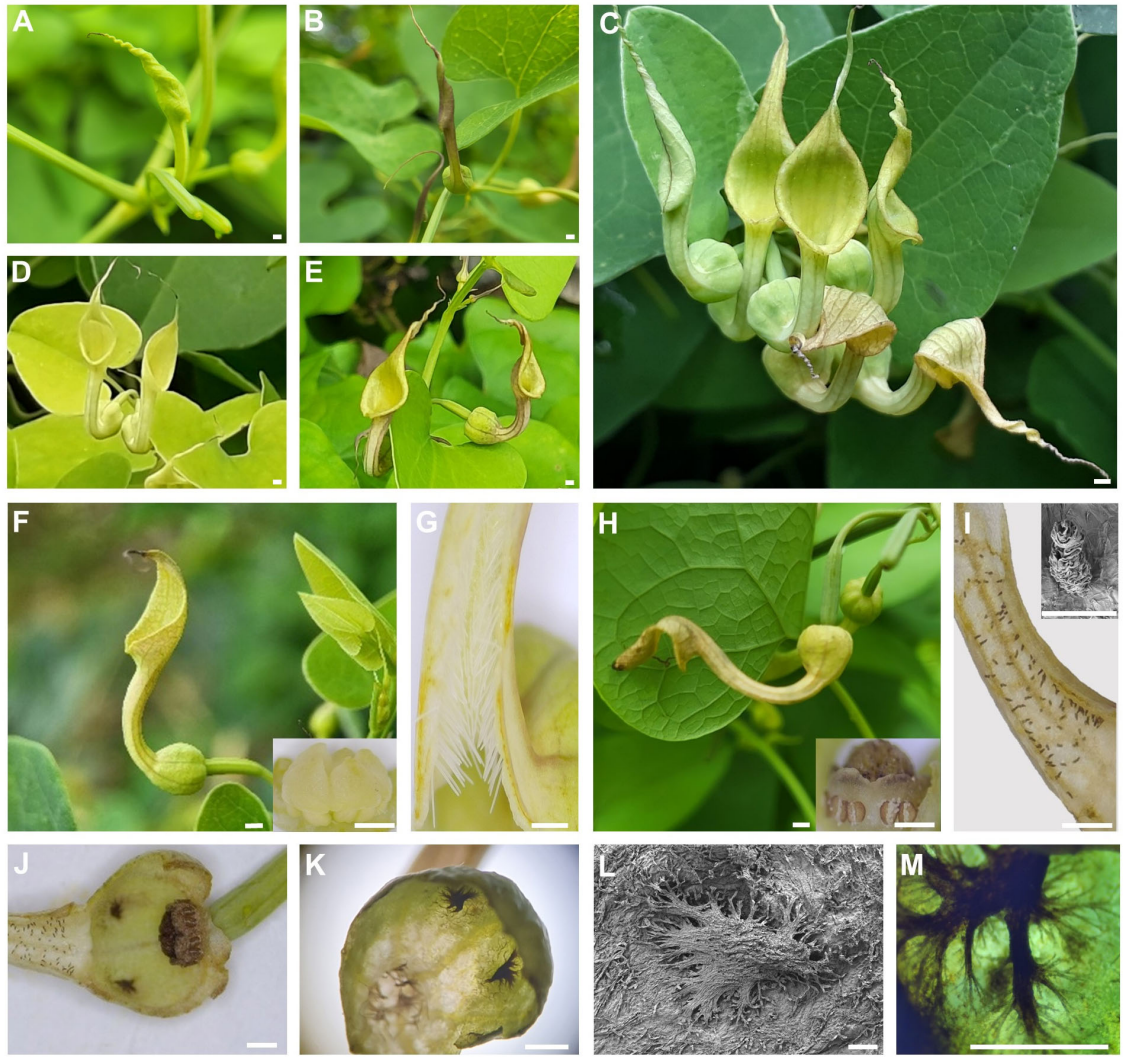
Microscopic examination of floral structures and phases of anthesis in *Aristolochia contorta.* **(A)** a yellow limb bud, **(B)** a purple limb bud, **(C)** inflorescences by order, **(D)** a yellowish perianth, **(E)** a purplish perianth, **(F)** a flower and gynostemium at the female phase of anthesis, **(G)** trichomes in the tube at the female phase of anthesis, **(H)** a flower and gynostemium at the male phase of anthesis, **(I)** trichomes in the tube at the male phase of anthesis, **(J)** inner utricle (side), **(K)** inner utricle with glands, **(L)** glands (scanning electron microscopy), **(M)** glands (optical microscope). The scale bars of **(A–K)** = 1 mm, and the scale bars of (the inset **I**), **(L, M)** = 100 µm.

*Aristolochia contorta* had long twining stems that used approximately 200 cm tall shrubs as support for climbing. Flowers bloomed first in the leaf axils and then began to bloom clockwise (**Figure 2C**). The perianth stood upright with the entrance upward, and then gradually bent down as it withered. The flowers of *A. contorta* had yellowish or purple perianths with brownish venation (**Figures 2A, B, D, E**). As the utricle expanded, the green buds became brighter green, creating a darker gradient toward the limbs. The tubular calyx has three distinct parts: limb, tube, and utricle. The yellowish or purplish two-lobed limb opened with the end twisted and was then unwound on the opposite side (**Figures 2A, B**). Trichomes, which extensively covered the inner surface of the perianth tube during the female phase of anthesis (**Figure 2G**), lost turgidity and contracted during the male phase (**Figure 2I**). In the utricle, the styles of the gynostemium were fused together with six stigmas and 12 stamens without filaments, two on each side of the style (**Figures 2F, H**). The six tips of the stigma lobes were narrowed and bent over the inside (**Figures 2F, H**). The upper side of the utricle, close to the tube, was white, with two or more dark secretory glands (Hipólito et al., 2012; **Figures 2J-M**). Fungi were sometimes observed in the glands.

The flowers were protogynous, and the floral anthesis of *A. contorta* had two distinct phases. After the flower opened, the first (female) phase lasted for 2–3 days (**Figure 2F**). The stigma lobes were receptive to pollen and were covered by a secretion for three days after the flower opened; during this time, the anthers were closed (**Figure 2F**). The stigma lobes lost their receptivity, and the anthers opened during the second (male) phase of anthesis, which began 3–4 days after flower opening (**Figure 2H**). Measurements were taken of the whole flower and each flower part from ten individual plants (**Table 1**).

**Table 1:** Measurements of the whole flowers and flower parts of each selected flower from 10 individual plants (n=10).

Measurement (length)	Value (mm, mean ± SE)
Whole flower	26.43 ± 1.19
Perianth tube	10.76 ± 0.99
Smallest diameter of perianth tube	2.64 ± 0.90
The widest diameter of the utricle	5.25 ± 0.12

### 3.2 Insect visitation

We identified 24 individual flower visitors by video recording, representing 8 species (**Table 2**). There were visitors that went into the flower and came out right away (referred to as the “free entry” group, denoted as F in **Table 2**), and there were those who went inside the flower and were trapped, and did not re-emerge during the recording time (referred to as the “trapped” group, denoted as T in **Table 2**). The average number of visitors per flower recorded during the monitoring period was 0.26 per hour. While aphids, mites, and ants were observed visiting the flowers in our study, they typically departed within 10 s.

**Table 2:** Flower visitors observed during video recordings of *Aristolochia contorta*. The number in parentheses is the number of flower visitors.

	Directly post-sunrise	Daytime	Directly post-sunset
**Jun. 18**	*Ponticulothrips diospyrosi* (1, F)	Tetranychoidea sp. (1, F)	–
**Jun. 25**	*Frankliniella occidentalis* (2, F)	–	Ceratopogonidae sp. (1, T)
**Jul. 02**	*Frankliniella occidentalis* (2, F), Ceratopogonidae sp. (1, T)	*Phintella* sp. (1, F)	–
**Jul. 09**	*Myzus persicae* (1, F), *Phintella* sp. (1, F), *Frankliniella occidentalis* (2, F)	*Frankliniella occidentalis* (2, F), Formicidae sp. (carrying Ceratopogonidae sp.) (1, F)	*Frankliniella occidentalis* (1. *F*)
**Jul. 16**	*Frankliniella occidentalis* (1, F), Formicidae sp. (1, F)	–	Formicidae sp. (1, F)
**Jul. 23**	Formicidae sp. (1, F)	–	*Frankliniella occidentalis (1, F)*
**Jul. 30**	–	–	–
**Aug. 06**	–	–	*Drosophila* sp. (1, F)
**Aug. 13**	–	Ceratopogonidae sp. (1, F)	–
**Aug. 20**	–	–	–
**Aug. 27**	–	–	–

F, “free entry” group; T, “trapped” group; -, no visitor.

**Figure 3:**
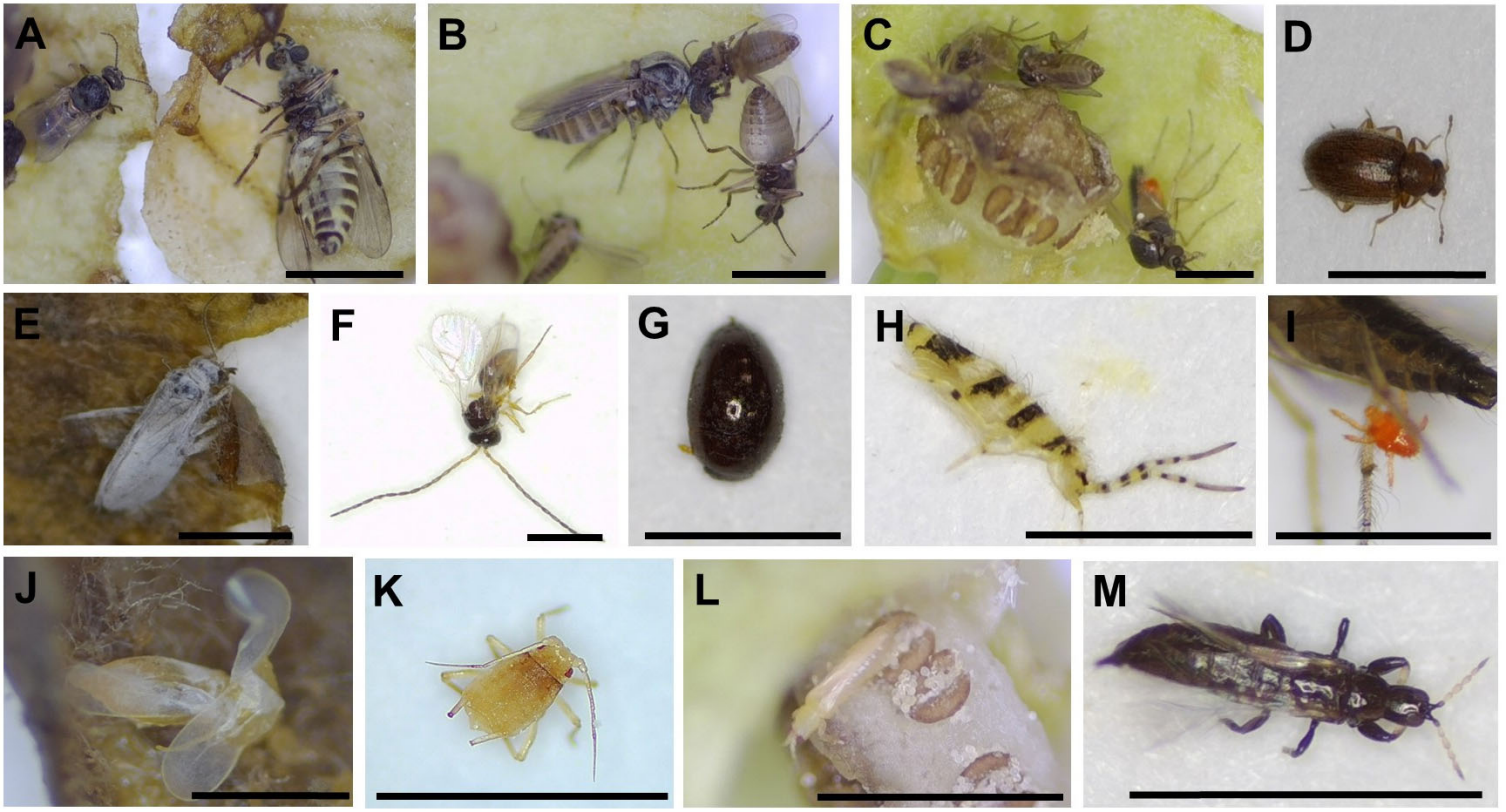
Diverse visitors trapped in *Aristolochia contorta* flowers. **(A–C)** Ceratopogonidae spp., **(D)** Cicadellidae sp., **(E)** Psocoptera sp., **(F)** Braconidae sp., **(G)** *Scymnus* sp. **(H)** *Entomobrya vigintiseta*, **(I)** Tetranychoidea sp., **(J)** Typhlocybinae sp., **(K)** *Myzus persicae*, **(L)** *Frankliniella occidentalis*, **(M)** *Ponticulothrips diospyrosi*. All the scale bars = 1 mm.

**Figure 4:**
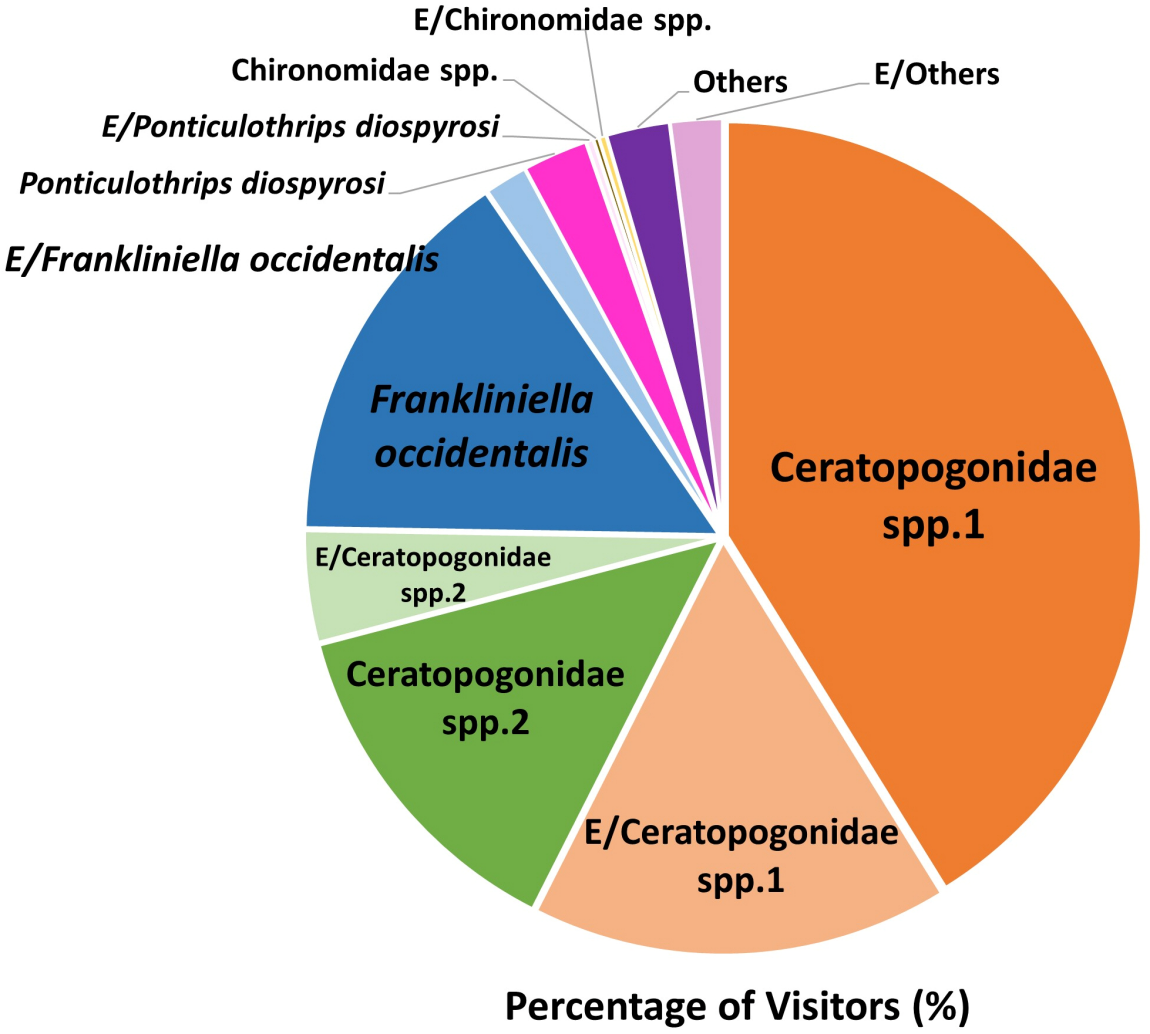
Percentage of the species of visitors to the *Aristolochia contorta* flowers while investigating the pollinators of the plant. E/: Percentage of visitors exited from flowers in a bottle.

We found 14 taxa of insects or arthropods (a total of 1,375) trapped in 473 out of 4,558 flowers after opening the flowers under a stereoscopic microscope (**Figure 3**). Although various species of visitors were trapped in flowers, Ceratopogonidae spp. accounted for the largest proportion of flower visitors (**Figure 4**). Ceratopogonidae spp. accounted for 75.27%, followed by *Frankliniella occidentalis* (16.87%), *Ponticulothrips diospyrosi* (2.84%), Chironomidae spp. (0.51%), and others (4.51%). 20.65% of Ceratopogonidae spp., 1.67% of *F. occidentalis*, 0.29% of *P. diospyrosi*, 0.29% of Chironomidae spp, and 2.04% exited from flowers (**Figure 4**).

**Figure 5:**
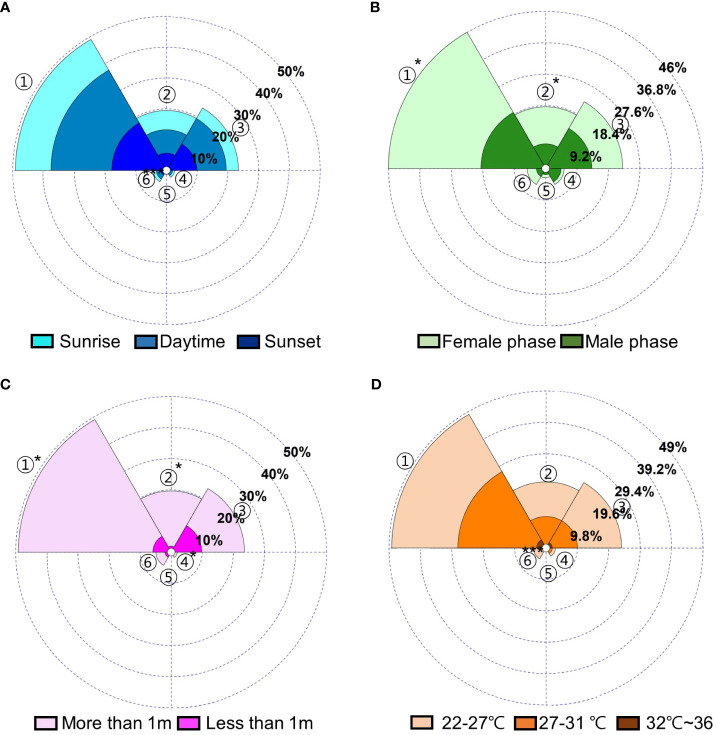
Distributions (%) of visitors to *Aristolochia contorta* according to **(A)** the time of day, **(B)** different developmental stages, **(C)** height, **(D)** temperature. 1. Ceratopogonidae spp. 1, 2. Ceratopogonidae spp. 2, 3. *Frankliniella occidentalis*, 4. *Ponticulothrips diospyrosi*, 5. Chironomidae spp., 6. others. * p < 0.05, *** p < 0.001.

There was a significant difference in the number of Ceratopogonidae spp. observed at different developmental phases and heights (Ceratopogonidae sp. 1, Ceratopogonidae sp. 2. p<0.05, respectively); however, there was no significant difference observed as a result of the time of day or temperature (**Figure 5**). In addition, there were no interactive effects among the time of day, different developmental stages, height, or temperature in the number of Ceratopogonidae spp. (**Table 3**).

**Table 3:** Multivariate ANOVA of the number of visitors to *Aristolochia contorta* according to the time of day, different developmental stages, height, and temperature.

	Time	Developmental phase	Height	Temperature	Time×Developmental phase	Time× Height	Time× Temperature	Height× Developmental phase	Height× Temperature	Temperature× Developmental phase	Time× Height× Developmental phase	Time× Height× Temperature	Height× Temperature× Developmental phase	Time×Temperature× Developmental phase	Time×Height ×Temperature ×Developmental phase
**Ceratopogonidae sp.1**	0.537	**4.004**	**3.427**	0.412	0.583	0.839	0.452	1.891	2.151	0.346	0.515	0.410	0.359	0.411	0.384
**Ceratopogonidae sp.2**	0.748	**4.775**	**3.808**	0.700	1.341	2.127	0.098	1.327	1.824	0.960	1.924	0.237	0.384	0.192	1.283
***Frankliniella occidentalis***	1.982	0.181	0.103	2.346	**3.523**	**5.399**	0.045	0.131	**3.728**	0.801	**4.007**	1.584	1.151	1.768	**2.753**
***Ponticulothrips diospyrosi***	1.009	1.268	**4.104**	1.731	2.884	3.798	2.800	**4.324**	**5.869**	2.204	**4.427**	**4.023**	**3.495**	**3.865**	**5.474**
**Chironomidae spp.**	5.449	2.694	2.215	0.106	1.605	**19.123**	0.689	**3.448**	0.184	0.424	**6.165**	**14.787**	2.098	1.070	**4.308**
**Others**	**16.086**	0.786	0.561	**19.344**	**19.586**	**36.661**	**19.779**	0.032	**35.843**	**15.901**	**41.871**	**32.803**	**8.377**	**19.745**	**34.446**

F statistics are shown. df = 3, 4554 for traits. Significant effects are shown in boldface (p < 0.05).

Ceratopogonidae spp., a potential pollinator known to carry pollen grains, exhibited a size range of 1.4 to 2.3 mm in length and 0.5 to 1.0 mm in width within the flowers of the female and male phases, respectively.

### 3.3 Pollination experiment

**Figure 6:**
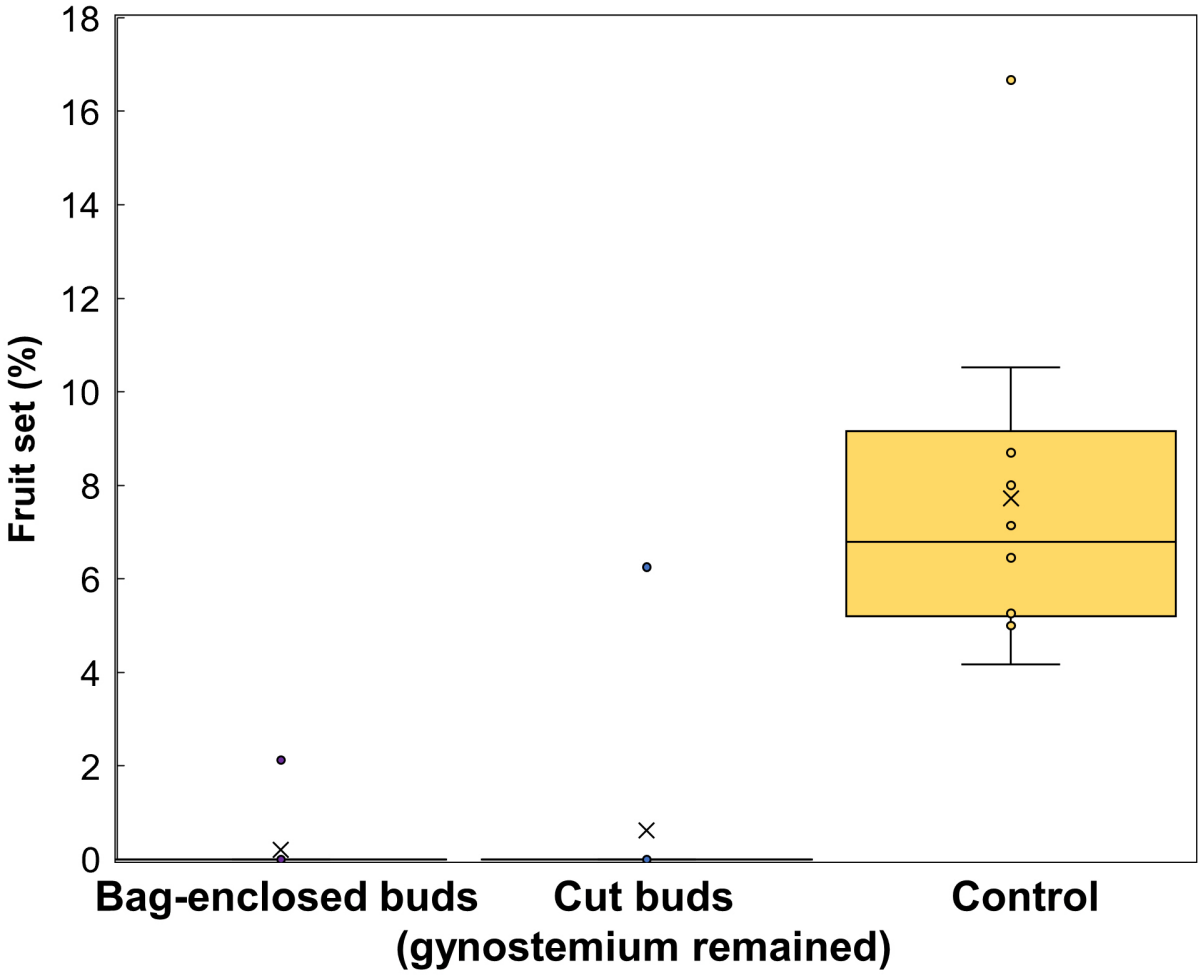
The percentage of fruit set in the three groups of *Aristolochia contorta*, including flowers that were enclosed in bags, buds that were cut, and the control.

The percentage of fruit set in bag-enclosed flowers and cut buds was significantly lower than that in the control group (p<0.05; bag-enclosed flowers, 0.2%; cut buds, 0.6%; control group, 7.7%; **Figure 6**). Even though few of bag-enclosed flowers and cut buds made fruit set, they were tiny aborted fruit. Therefore, we confirmed that *A. contorta* is an allogamous and entomophilic plant, consistent with other members of the Aristolochiaceae.

### 3.4 Pollen distribution on pollinator bodies

**Figure 7:**
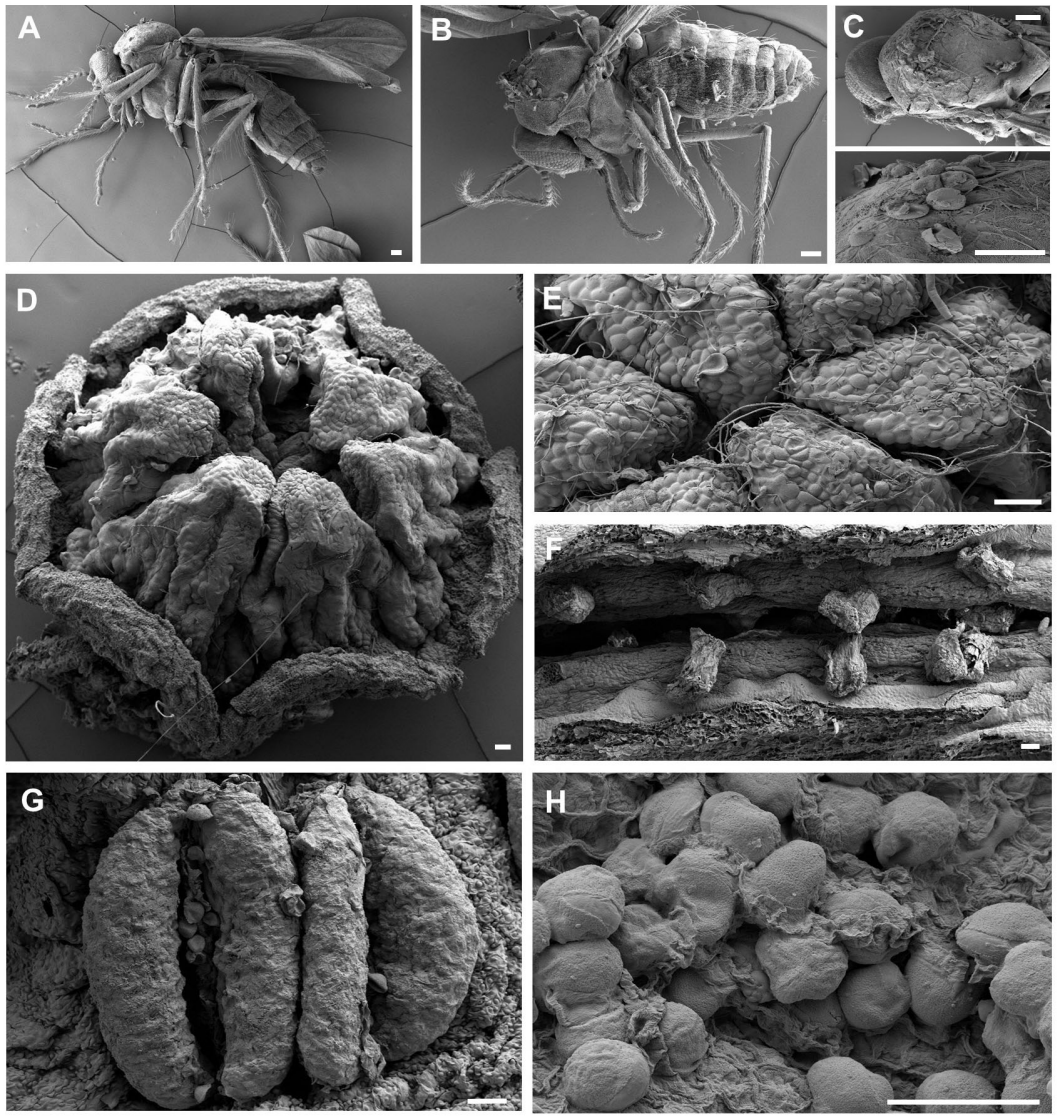
Visual evidence of the distribution and transfer of pollen grains on the floral reproductive structures and the bodies of potential pollinators, **(A)**, **(B)** Ceratopogonidae spp., **(C)** Ceratopogonidae sp. with pollen on their thoraxes, **(D)** upper side of gynostemium (stigma), **(E)** pollen grains and their tubes on the stigma (pollination), **(F)** seeds developing in the ovary (fertilization). **(G)** stamens on the side of an *Aristolochia contorta* gynostemium, **(H)** *A. contorta* pollen grains in the utricle. All the scale bars = 50 μm.

**Figure 8:**
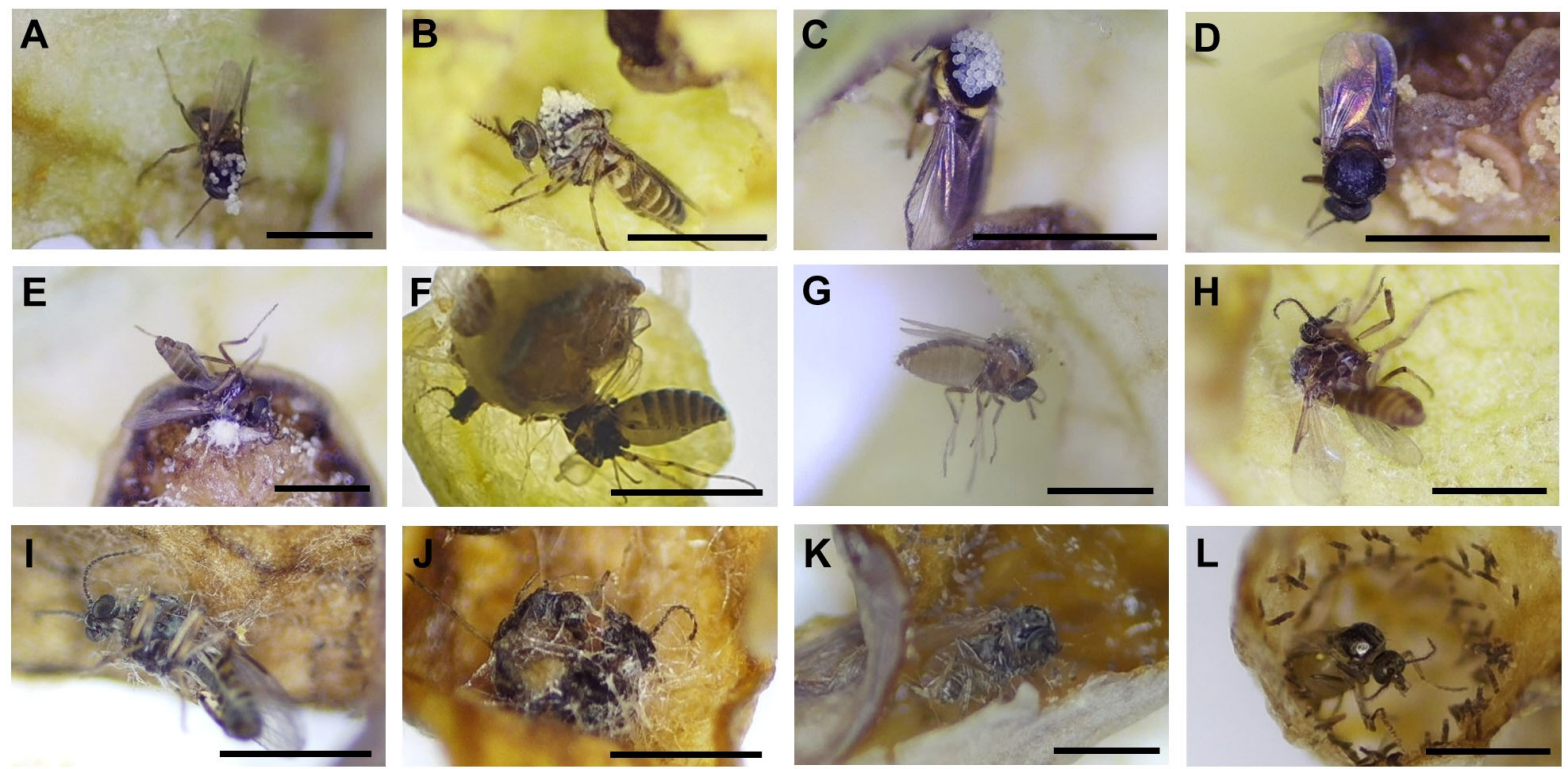
Pollen-loaded floral visitors and trapped visitors **(A–D)** Ceratopogonidae spp. with pollen, **(E, F)** Ceratopogonidae spp. trapped to death on the gynostemium, **(G–J)** Ceratopogonidae spp. trapped to death on the utricle, **(K, L)** Ceratopogonidae spp. trapped to death on the tube. All the scale bars = 1 mm.

During the male phase in the flower, pollen grains adhered to the visitors’ bodies. However, only Ceratopogonidae spp., which had hairs on the dorsal surface of the thorax (**Figures 7A-C**), were found to have attached pollen grains (**Figures 8A-D**). Of the 948 Ceratopogonidae spp., 248 individuals (26.16%) had loaded pollen grains. Among the 473 flowers with visitors, 287 contained Ceratopogonidae spp. with an average of 3.31 individuals per flower. Although Ceratopogonidae spp. were loaded with pollen grains (an average of 54.24 pollen grains were attached), most of them died in the flowers (exit rate, 21.76%). They either failed to exit the tube or became stuck to the sticky upper part (stigma) of the gynostemium or the sticky interior of the perianth tube and utricle (**Figures 8E-L**). Out of the 248 individuals of Ceratopogonidae spp. with attached pollen grains, 173 died while still attached to the inner part of the perianth (69.76%, **Figures 8E-L**).

SEM observations revealed the distribution of pollen grains in both flowers and Ceratopogonidae spp. Pollen grains were found on the stigma (the upper surface of the gynostemium) and stamen (the lateral surface of the gynostemium) (**Figure 7**). Additionally, they were observed on the thorax of hairy Ceratopogonidae spp. that entered the perianth.

## 4 Discussion

### 4.1 *Aristolochia contorta*, an allogamous species pollinated by Ceratopogonidae spp.

In our pollination experiment (**Figure 6**), we verified that *A. contorta* is an allogamous plant. The SEM observations provided visual evidence of the distribution of pollen grains in flowers and Ceratopogonidae spp. The presence of pollen grains on both the stigma and the stamen suggested that pollination has occurred, provided that the pollen grains on the stigma did not belong to the same flower. Additionally, the presence of pollen grains on the thorax of hairy Ceratopogonidae spp. indicated that these insects have come into contact with the flower’s reproductive structures while entering the perianth. This suggested that the Ceratopogonidae spp. might be acting as pollinators for these flowers. Furthermore, these findings provided additional information on the specific interactions between *A. contorta* flowers and pollinators. This discovery supports earlier research on pollination mechanisms in *Aristolochia* species, which also depend on dipteran pollinators (Berjano et al., 2011; Hipólito et al., 2012; Aliscioni et al., 2017; Martin et al., 2017).

### 4.2 Insect attraction

During the monitoring period, we observed an average of 0.26 visitors per flower per hour. This value is lower in comparison to other *Aristolochia* species, such as *A. baetica* (0.39 visitors/flower/h; Berjano et al., 2011), *A. chilensis* (0.4-0.6 visitors/flower/h; Valdivia and Niemeyer, 2007), *A. pilosa* (2.1-6.0 visitors/flower/h; Wolda and Sabrosky, 1986), and *A. littoralis* (3.6 visitors/flower/h; Hall and Brown, 1993). Only 11.57% (473 out of 4,558 flowers) of the flowers contained insects and *A. contorta* primarily attracted Diptera (Ceratopogonidae spp. accounted for 75.27% among insects), which is consistent with other *Aristolochia* species (Sakai and Disney, 2001; Burgess et al., 2004; Trujillo and Sérsic, 2006; Rupp et al., 2021; Alpuente et al., 2023). Flowers frequently visited by Diptera usually have nectar dominated by hexoses as the main sugar (Kevan and Baker, 1983; Galetto and Bernardello, 2003). We acquired 73 hours of footage over a period of 10 weeks, but only three Ceratopogonidae spp. entered the flowers. In addition, a large number of Ceratopogonidae spp. were concentrated in a single flower (up to 14 individuals). These observations suggest that while the overall attraction of Ceratopogonidae spp. to the flowers may be relatively low, certain flowers exhibit a high level of attraction and act as focal points for gathering these insects. Several *Aristolochia* species secrete nectar (Vogel, 1998; Trujillo and Sérsic, 2006; Erbar, 2014), which helps pollinators sustain their survival when trapped in the utricle (Vogel, 1998; Sakai, 2002; Trujillo and Sérsic, 2006; Ollerton, 2021). In *A. contorta*, as in other species, the glands and pollen grains, which are sticky and moldy, may play a role in providing food for Diptera.

### 4.3 Trapping

The structure of *A. contorta* flowers, which has a narrow tube-like perianth, is suitable for pollination by tiny flying insects (Berjano et al., 2009; Hipólito et al., 2012). For pollination, the pollinator must move freely from the outside to the inside of the flower, and vice versa. There are significant relationships between pollinator size, in terms of either length or width, and the smallest diameter of the perianth tube (Burgess et al., 2004; Trujillo and Sérsic, 2006; Rulik et al., 2008). Furthermore, *A. contorta* exhibits several adaptive traits that make it suitable for pollination by Ceratopogonidae spp. due to the specific characteristics of its flower structure. The perianth tube is narrow (2.64 ± 0.90 mm), and the utricle is relatively small (diameter 5.25 ± 0.12 mm), which is suitable for the size of Ceratopogonidae spp. (1.4 to 2.3 mm in length, 0.5 to 1.0 mm in width) compared to larger insects. Additionally, *A. contorta* flowers possess a vertical perianth tube structure that enables efficient contact with the utricle, resulting in the entrapment of insects. Trichomes present on *A. contorta* flowers further contribute to physically trapping insects, serving as a protective barrier (Gorb and Gorb, 2002). This is similar to other *Aristolochia* species, in which trichomes within the perianth are oriented in the utricle, naturally guiding insects downward (Oelschlägel et al., 2009; González and Pabón-Mora, 2015; Rupp et al., 2021). The reduction of tube width caused by a dense trichome arrangement prevents insects move sideways (Guimarães et al., 2008). Thus, the vertical perianth tube and the trichomes in the tube can trap insects in flowers. These are the two main trapping mechanisms that have evolved, and one or both can be found in all pitfall flowers, including those of the genera *Aristolochia* and *Ceropegia* (Masinde, 2004), as well as *Sarracenia purpurea* (Tagawa, 2020).Therefore, our study corroborated previous research on the trapping mechanism of *Aristolochia* and contributed to a comprehensive understanding of the adaptations exhibited by *A. contorta* for optimal pollination, as well as the distinctive attributes of its floral structure.

### 4.4 Retention

During our observations, we identified several species that were less affected by trichomes, but not all of them were effective pollinators. Some *Aristolochia* species have adapted to trap Diptera of specific sizes (Brantjes, 1980; Rupp et al., 2021) or other particular insect species (Razzak et al., 1992; Rupp et al., 2021; Alpuente et al., 2023). Among the many visitors, the small size (1 mm) and smooth body of the Diptera made it easy to avoid the trichomes. In our study, *F. occidentalis* and *P. diospyrosi* entered the flowers and did not emerge within one hour of recording. Consequently, *A. contorta* may be selected as a brood or breeding site, since pollen was not observed on their bodies; therefore, they were excluded from the study as potential pollinators. Previous research also assumed that it is a pollen feeder that develops on living flowers and rarely aids in pollination (Mound and Marullo, 1996; Bolin et al., 2009; Peris et al., 2020; Das and Das, 2023). Ceratopogonidae spp. were the most common visitors to *A. contorta*, constituting 71.5% of the total number of insects collected from inside the flowers. The insects were unable to escape because the trichomes trapped them and kept them inside the utricles. This is because significant effort is required to exit the flower during the female phase, which may not be achievable by insects of this size (Razzak et al., 1992). Therefore, the morphological and biomechanical characteristics of trapping trichomes represents a highly effective method for trapping and retaining insects.

We observed that many pollinators trapped in the female phase of *A. contorta* flowers transitioned to the male phase during their prolonged stay inside the flowers. As the anthers opened, pollen grains were released and landed on the hairy dorsal thorax of the Ceratopogonidae spp. (**Figure 7C**). The hairy bodies of these pollinators facilitated the collection of pollen grains (**Figure 7A-C**), and the dark glandular spots within the utricle may have served as guides, aiding the adherence of pollen grains to the thorax (Dafni et al., 2005). The pollen of *A. contorta* was also sticky, further promoting its attachment to pollinator bodies.

The exact number of pollen grains required for fertilization may vary depending on factors such as the effectiveness of the pollinators, the genetic makeup of the plant, and environmental conditions (Shivanna and Rangaswamy, 2012; Nakonechnaya et al., 2021). We observed 12 anthers and a total of 1800 pollen grains in each of *A. contorta*’s flowers and this species has 24.54 ± 2.98% of pollen grains that were defective (Nakonechnaya and Kalachev, 2018). In our observations, the minimum number of viable seeds per fruit was approximately 60 (a fruit contained 152.2 ± 5 seeds), so this number of ovules should be successfully fertilized for the ovary to develop into a fruit. Therefore, considering the defectivity ratio of pollen grains and the minimum number of seeds per fruit, visitors should load at least 75 pollen grains for the fertilization of a single *A. contorta* flower. We observed that small Ceratopogonidae spp. (2–3 mm) carried an insufficient number of pollen grains, with an average of 54.24 per individual. Ceratopogonidae spp. tended to concentrate on specific flowers with considerable variation, ranging from 0 to 14 individuals per flower (Ceratopogonidae sp. 1. averaged 2.08 ± 0.24 individuals per flower, while Ceratopogonidae sp 2. averaged 1.32 ± 0.18 individuals per flower. The trapped Ceratopogonidae spp. were retained together in the utricle, with an average diameter of 5.25 mm ± 0.12. Although we observed mating in one instance, we could not confirm whether the eggs were laid on the flower. Furthermore, we noted that the trapped Ceratopogonidae spp. remained alive inside the flowers for up to 15 days, suggesting that *A. contorta* may serve as a brood or breeding site. However, this prolonged stay inside the flower could result in pollination failure, as the pollen grains on their bodies may lose their vitality over time. The viability of pollen grains can be influenced by various factors, including genetics, climatic conditions, the timing of collection, and the duration between collection and pollination (Percival, 2013). Among these factors, the retention time or the time spent inside the flower may play a role in pollen vitality. This knowledge contributes to our understanding of the reproductive success *of A. contorta* and the role of pollinators in ensuring successful fertilization.

### 4.5 Release

To ensure successful pollination, pollen-laden insects must exit and enter the tubes of other flowers. However, our observations revealed a low exit rate (21.76%) of pollinators from *A. contorta* flowers, indicating that trapping was effective, but the release was not. We observed that the vertical perianth tube and trichomes in the female phase prevented insects from leaving the flower, whereas in the male phase, insects were released as the perianth gradually withered downward and trichomes shrunk (**Figure 2I**; Vogel and Martens, 2000; Singer, 2002; Nishizawa et al., 2005). This could be due to special structural changes such as organ withering or alterations in surface texture. In *A. contorta*, the stiff white trichomes that were densely packed downward in the tube lost turgidity and contracted, creating an opening in the center of the tube when it entered the male phase to facilitate the exit of pollinators and pollen transport (**Figure 2I**). Similar changes in trichome directionality and flower position have been observed in other *Aristolochia* species and may contribute to the trapping and release of pollinators (Burgess et al., 2004; Oelschlägel et al., 2009; Kidyoo et al., 2022). Trichomes of *Aristolochia* flowers play a significant role in the capture, retention, and release of insects. However, the internal cavity of the perianth has a sticky texture, which often results in adhesion and clogging of the narrow perianth tube (2 mm in diameter). In addition, we noted that 12.33% of Ceratopogonidae spp. were found adhered to the perianth wall and had already died when we manually opened the flowers. Moreover, the size and force of Ceratopogonidae spp. may limit successful pollination (Dafni et al., 2005). Consequently, many pollinators were unable to escape due to the flower’s characteristics, leading to reduced reproductive success.

### 4.6 Perianth necessary for pollination causes low pollination effectiveness

Our study examined the role of floral traits, as well as the impact of pollinator availability and efficiency, on the reproductive success of *A. contorta*. Most flowers are designed to attract a wide range of pollinators, including bees, butterflies, and other insects (Kevan and Baker, 1983; Thompson, 2001). These flowers typically have bright colors, attractive volatiles, and easily accessible nectar and pollen (Varassin et al., 2001; Dobson, 2006). In contrast, the unique shape of *Aristolochia* flowers specializes in attracting and trapping a specific group of pollinators and trap them. Structurally, the perianth of *Aristolochia* flowers traps pollinators with the help of trichomes, which densely cover the inner surface of the perianth tube during the female phase. The U-shaped tube of the flower, which expands at the terminal end, is equipped with various trap-and-release mechanisms such as trichomes (**Figures 2G, I**) to maintain insects inside the flower until the anthers burst. Some *Aristolochia* species are specifically designed to trap Diptera of a particular size or even a specific species of insect (Brantjes, 1980; Razzak et al., 1992; Rupp et al., 2021; Alpuente et al., 2023). Therefore, the pollination experiments (**Figure 6**), in which only the gynostemium was retained and the perianth (buds) was excised, along with previous studies on *Aristolochia* flowers have shown that the perianth plays a crucial role in pollination.

**Figure 9:**
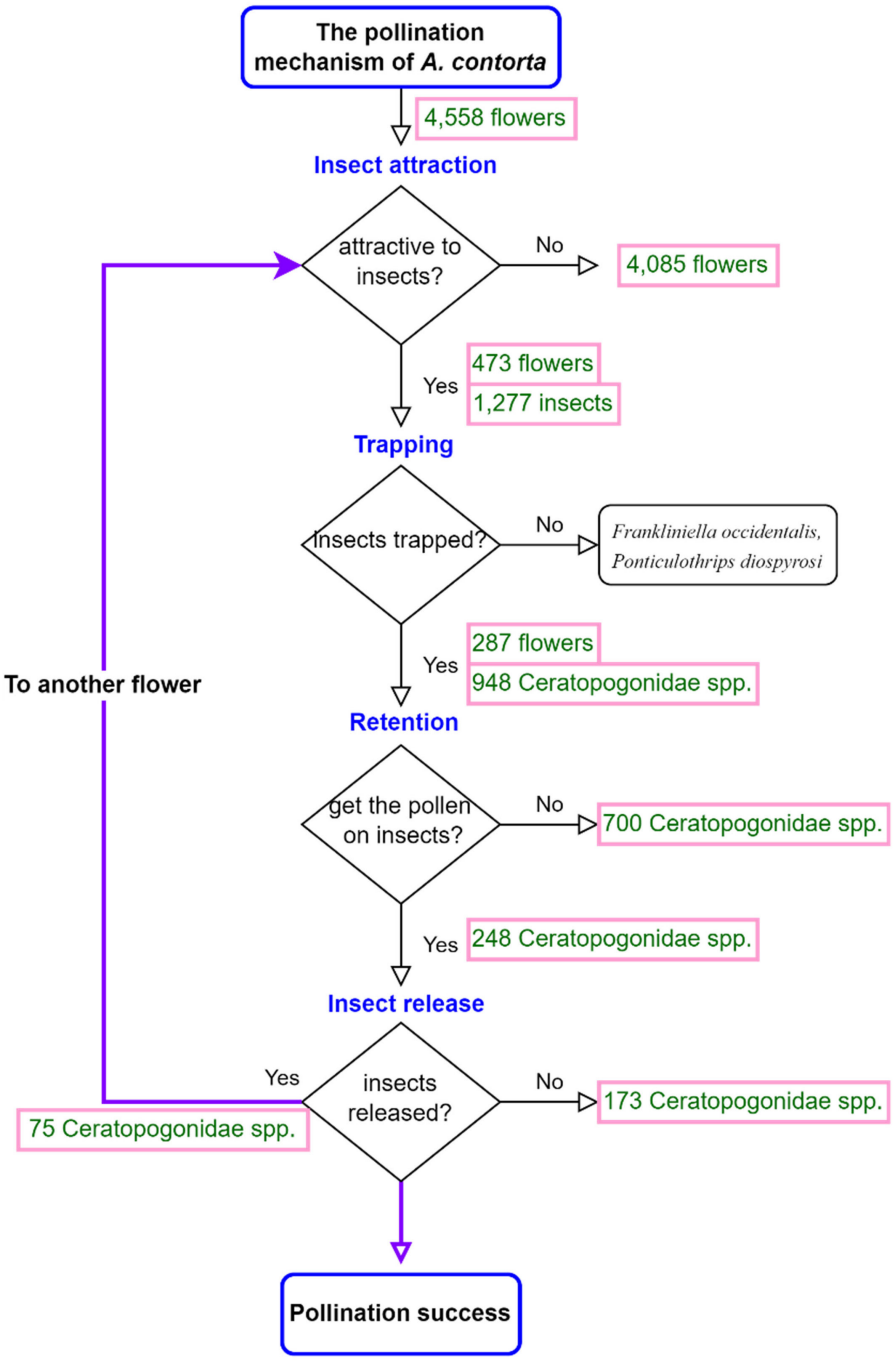
The pollination mechanism of *Aristolochia contorta.* The numbers in the flowchart indicate the number of insects or flowers that answered Yes or No to the question.

However, in a paradoxical twist, the perianth’s effective trapping mechanism also results in an unintended consequence: a 12.33% mortality rate among pollinators. As a result, the overall pollination effectiveness of *Aristolochia* flowers remains relatively low (**Figure 9**). This could be attributed to the floral trait of the perianth which is a sticky texture of the internal cavity. This sticky texture may pose a challenge for certain pollinators when interacting with the flower, potentially affecting their ability to effectively transfer pollen and contribute to successful pollination (Sakai, 2002). Low fruit set (fruit set ratio of *Aristolochia*: *A. rotunda*, 12.7%; *A. paucinervis*, 25%; *A. manshuriensis*, 2%; *A. baetica*, 4-14%; *A. inflata*, 18.7%; *A. maxima*, 2.4%) seems to be a general phenomenon in *Aristolochia* (Sakai, 2002; Berjano et al., 2006; Murugan et al., 2006; Trujillo and Sérsic, 2006; Nakonechnaya et al., 2021).

## 5 Conclusion

*Aristolochia contorta* possesses several adaptations that deter inbreeding and foster the maintenance of genetic diversity, including a protogynous system and pollination through the trapping of pollinators. Furthermore, the plant generates a substantial quantity of flowers sequentially over an extended flowering period, resulting in a notable seed yield per fruit. Despite evolving into a highly intricate and specialized pollination mechanism and exhibiting abundant flower production, it is apparent that this strategy falls short in achieving adequate pollination, as evidenced by the low rate of effective pollination observed in our study. This highlights that despite the significant energy and resources invested in flower development, there are constraints regarding successful pollination, which can be related to the densely clustered distribution of its pollinators in only a few flowers, insufficient pollen loading onto pollinators, hindrance in the proper release of entrapped pollinators due to the adhesive surface of the perianth, and a high rate of defective pollen grains in *A. contorta*. Collectively, these factors contribute to the observed phenomenon of low fruit set.

Therefore, the low effective pollination, combined with its reproductive dependence on biotic vectors, may lead to rely heavily on asexual reproduction (producing new shoots from underground rhizomes), which could potentially lead to increased levels of genetic segregation. Furthermore, if the pollinators fail to consistently visit the flowers, the reproductive success of the plant can be greatly compromised. This diminished rate raises concerns about the long-term survival and adaptability of *A. contorta* in the face of changing environmental conditions. This study emphasizes the importance of conservation efforts and management strategies to ensure the preservation of this unique plant species. In the future study, investigating the behavior and preferences of Diptera pollinators, analyzing floral morphology and anatomy, and assessing genetic diversity and the risk of inbreeding depression are important avenues to explore. Additionally, developing conservation strategies and conducting comparative studies within the *Aristolochia* genus can provide insights into pollination mechanisms and reproductive strategies. These efforts will not only contribute to the conservation of *A. contorta* but also broaden our understanding of plant reproductive biology.

## Data availability statement

The original contributions presented in the study are included in the article. Further inquiries can be directed to the corresponding author.

## Author contributions

JGK, S-HP conceived the idea and designed the methodology; S-HP collected and analyzed the data; and S-HP wrote the manuscript. All authors contributed critically to drafts and approved the final manuscript for publication.
